# Improvement of Tissue Survival of Skin Flaps by 5α-Reductase Inhibitors: Possible Involvement of Nitric Oxide and Inducible Nitric Oxide Synthase

**DOI:** 10.6091/ibj.1408.2015

**Published:** 2015-04

**Authors:** Ali Asghar Karimi, Marjan Ajami, Yasin Asadi, Nahid Aboutaleb, Fazel Gorjipour, Roya Malekloo, Hamidreza Pazoki-Toroudi

**Affiliations:** 1 *Dept. of Physiology and Physiology Research Center, Faculty of Medicine, Iran University of Medical Sciences, Tehran, Iran; *; 2 *Physiology Research Center, Faculty of Medicine, Iran University of Medical Sciences, Tehran, Iran; *; 3 *Dept. of Food and Nutrition Policy and Planning Research, National Nutrition and Food Technology Research Institute, Faculty of Nutrition and Food Technology, Shahid Beheshti University of Medical Sciences, Tehran, Iran;*; 4 *Physiology Research Center, Semnan University of Medical Sciences, Semnan, Iran*

**Keywords:** Finasteride, Azelaic acid, Surgical flaps, Nitric oxide, Nitric oxide synthase Type II

## Abstract

**Background:**

Skin flap grafting is a popular approach for reconstruction of critical skin and underlying soft tissue injuries. In a previous study, we demonstrated the beneficial effects of two 5α-reductase inhibitors, azelaic acid and finasteride, on tissue survival in a rat model of skin flap grafting. In the current study, we investigated the involvement of nitric oxide and inducible nitric oxide synthase (iNOS) in graft survival mediated by these agents.

**Methods:**

A number of 42 male rats were randomly allocated into six groups: 1, normal saline topical application; 2, azelaic acid (100 mg/flap); 3, finasteride (1 mg/flap); 4, injection of L-N^G^-nitroarginine methyl ester (L-NAME) (i.p., 20 mg/kg); 5, L-NAME (20 mg/kg, i.p.) + azelaic acid (100 mg/flap, topical); 6, L-NAME (20 mg/kg, i.p.) + finasteride (1 mg/flap, topical). Tissue survival, level of nitric oxide, and iNOS expression in groups were measured.

**Results:**

Our data revealed that azelaic acid and finasteride significantly increased the expression of iNOS protein and nitric oxide (NO) levels in graft tissue (*P *< 0.05). These increases in iNOS expression and NO level were associated with higher survival of the graft tissue.

**Conclusion:**

It appears that alterations of the NO metabolism are implicated in the azelaic acid- and finasteride-mediated survival of the skin flaps.

## INTRODUCTION

Skin flap grafting is indicated for reconstruction of massive deep skin damages and underlying soft tissue injuries [1]. It is the most popular surgical method for critical skin defects especially when there is a need for massive reconstruction of complex anatomic structures, such as covering an amputation stump or reconstruction of breast [2]. The success rate of grafting is limited by ischemia/reperfusion (I/R)-induced injury of the graft tissue. Studies have revealed that microcirculatory intravascular thrombosis causes the I/R-induced morbidity of the flap that decreases the success rate of the procedure [3, 4]. 

Various approaches have been used to improve the outcomes and reduce the morbidities resulting from I/R injury of skin flaps [5, 6]. In addition, several pharmacological agents have been tested in preclinical [7, 8] and clinical studies [9]. Hence, there is a need for intensive studies for understanding the drug targets and agents applicable for the treatment of this situation. Dihydrotestosterone (DHT) has long been recognized for its detrimental effects on ischemic events [10, 11]. It has been known that prevention of DHT biosynthesis may improve the outcomes of I/R injury in animal models. We previously demonstrated that DHT synthesis inhibitors, azelaic acid and finasteride, preserve the skin flaps in rat models through reducing the apoptotic cell death of the skin flap [12]. Another study have also demonstrated DHT is responsible, at least in part, for the myocardial damage induced by I/R in male rats [13]. Beyond this, researchers have noted the vasoactive properties of DHT as a functional result of its effect on inducible nitric oxide synthase (iNOS) [14]. It is assumed that iNOS activation results in vasodilation and its local activation improve the blood supply of the affected tissue [15]. Thus, in the current study, we tried to investigate the potential involvement of nitric oxide (NO) metabolism in finasteride- and azelaic acid-induced survival of skin flaps in rat animal models. 

## MATERIALS AND METHODS

***Animals and ethical considerations. ***The study protocol was approved by Ethics Committee of the Iran University of Medical Sciences (Tehran, Iran). Male Sprague-Dawley rats (n = 42, weighing between 200 and 250 g), were chosen for random pattern cranial-based skin flap elevation. The animals were kept in separate cages with free access to food and water. 

***Experimental design. ***Experimental design, study groups, and surgical approaches to create models were according to our previous study [12]. Briefly, animals were allocated into six experimental groups: 1, normal saline topical application; 2, azelaic acid (100 mg/flap, Merck, USA); 3, finasteride (1 mg/flap, Reddy, India); 4, injection of L-N^G^-Nitroarginine methyl ester (L-NAME, 20 mg/kg, i.p., Sigma, Germany); 5, L-NAME (20 mg/kg, i.p.) + azelaic acid (100 mg/flap, topical); 6, L-NAME (20 mg/kg, i.p.) + finasteride (1 mg/flap, topical). In the last two groups, L-NAME was injected 30 minutes before azelaic acid or finasteride application. Drugs were solubilized in 10% (v/v) ethanol in distilled water. The solvent had no effect on the parameters studied in the current research. 

***Skin flap surgery and calculation of necrotic area. ***Method of surgical approach for creating skin flap models and calculation of necrotic area were performed based on the McFarlane's method [12]. Briefly, for calculating the necrotic area, photographs were taken from the flaps, and necrotic areas were measured by superimposition of photographs on a graph paper. The results were expressed as percentages of necrotic area and calculated as follows: the extent of necrotic area × 100/total area of the flap (viable and necrotic).

***iNOS protein expression in flap tissue. ***Western-blotting method was used to study the expression of iNOS protein in skin tissue of experimental groups. The protocol for preparation and Western-blot analysis of the samples were according to our previous experiment [12]. Rabbit anti-iNOS (1:200) and mouse anti-β-actin antibodies (both from Abcam, USA) were used in this study. 

***Measurement of nitrite/nitrate concentration in skin flaps. ***Preparation of tissue homogenate of skin flaps and control skin has been described previously [12]. Then the homogenate was centrifuged. After centrifugation, supernatant was removed and used to determine the levels of NO metabolites by assaying nitrite (NO2-) plus nitrate (NO3-) using the Nitric Oxide Colorimetric Assay Kit (Roche Applied Science, Indianapolis, IN, USA) according to the manufacturer's instruction. 

***Statistical analysis. ***SPSS statistical package (v. 17, IBM Corporation, USA) was used to analyze the data. Since the data showed a normal distribution pattern using Kolmogorov-Smirnov test as well as homogeneity of variance, the results were statistically evaluated by one-way analysis of variance (ANOVA). ANOVA was used for comparison of flap survival, iNOS/β-actin expression, and nitrite/nitrate concentration among multiple groups, followed by Tukey test for intergroup comparisons. Quantitative data were presented as mean ± SEM, and *P* value less than 0.05 was considered statistically significant.

## RESULTS AND DISCUSSION

**Fig. 1:**
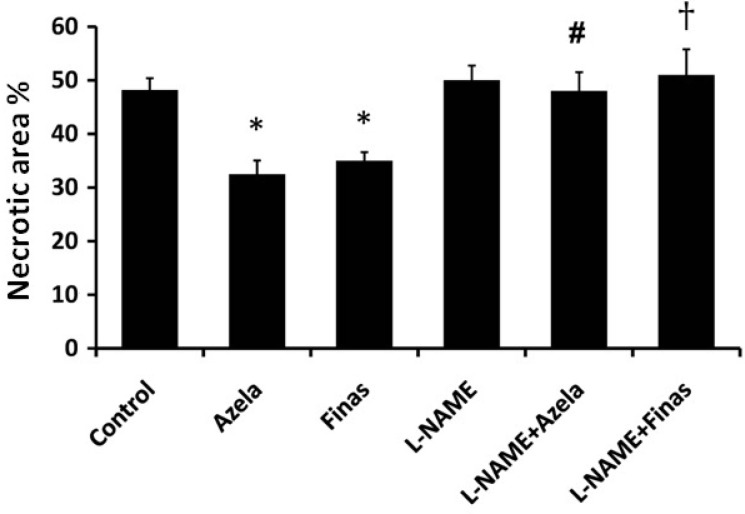
Effects of different type of treatments on skin flap survival. Data are shown as Mean ± Standard error of mean (SEM) of percent of necrotic area for each group. ^*^P < 0.05 vs. control group, ^#^*P *< 0.01 vs. azelaic acid, and ^†^*P* < 0.001 vs. finasteride. No significant differences between L-NAME, L-NAME+ Azela, L-NAME + Finas and the control group were observed. Azela, azelaic acid; Finas, finasteride.

In the current study, we demonstrated that topical administration of azelaic acid or finasteride improved the surgical flap survival and decreased graft tissue necrosis in a male rat model (Fig. 1). In control group, mean flap necrotic area was 48.17% ± 2.22%, which was significantly decreased to 32.5% ± 02.55% and 35.0% ± 1.59% (*P* < 0.05 vs. control group) after treatment by azelaic acid and finasteride, respectively. L-NAME by itself had no significant effect on flap necrosis (50% ± 2.75%, *P* > 0.05 vs. control group). 

**Fig. 2:**
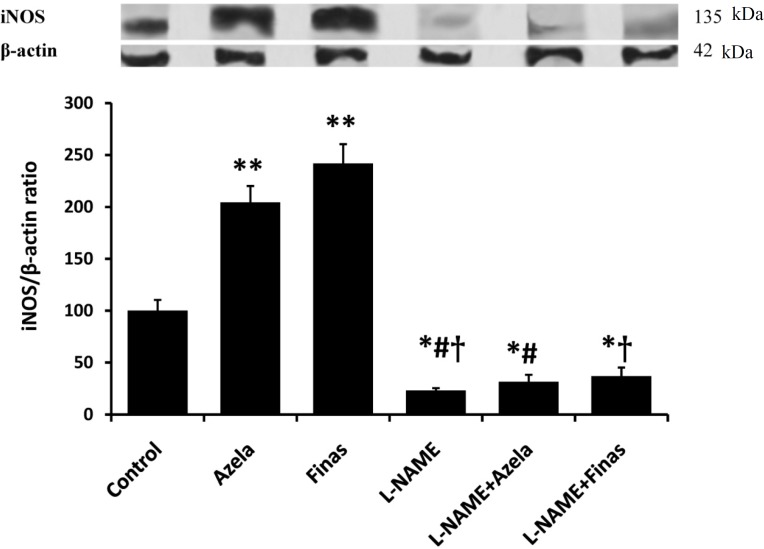
Expression of iNOS in skin flaps obtained from different groups of treatment. Relative densities of bands were normalized to the density of corresponding bands for β-actin and values represent means ± Standard error of mean (SEM) of each group. Upper panel illustrates a sample of iNOS and related β-actin expressions in each group. Lower panel shows the normalized values of iNOS/β-actin expression ratio. ^*^P < 0.05 and ^**^P < 0.01 vs. control group, ^#^*P* < 0.001 vs. azelaic acid and † *P* < 0.001 vs. finasteride. Azela: azelaic acid, Finas: finasteride.

However, in the animals treated with azelaic acid, pre-treatment with L-NAME increased the average necrotic area to 48.0% ± 3.52% (*P* < 0.01), which was significant when compared to the group with azelaic acid only. Similarly, in the case of finasteride, pre-treatment with L-NAME increased necrotic area (*P *< 0.001 vs. finasteride alone). These results presumably demonstrate the NO-dependent mechanism of effect of azelaic acid and finasteride on survival of surgical flap. 

Treatment with azelaic acid or finasteride increased iNOS expression in skin tissue in comparison to control group (*P* < 0.01, Fig. 2). iNOS/β-actin ratio was increased more than two-fold when skin flaps were treated with azelaic acid and finasteride. While it is reasonable to consider this increase of iNOS expression as a result of DHT synthesis inhibition, existing data on iNOS expression in response to DHT treatment from different studies are versatile. Gonzalez *et al.* [15] have noted the elevation of iNOS expression level in cerebral artery endothelium following * ex vivo* DHT treatment. Bae *et al.* [16] have reported that *in vivo* treatment of rat vascular smooth muscle cells with DHT significantly decreased the iNOS protein expression. On the other hand, Kolasa *et al.* [17] have indicated that DHT can regulate the expression of iNOS, and finasteride-induced DHT deficiency upregulates iNOS expression [14]. 

Surprisingly, treatment with L-NAME significantly decreased iNOS expression compared to the control group (*P *< 0.05). Moreover, pre-treatment with L-NAME decreased iNOS expression in flaps treated with azelaic acid and finasteride compared to the control groups (*P* < 0.05) having azelaic acid- and finasteride-treated flaps alone (*P* < 0.001, Fig. 2). To date, no direct effect of L-NAME on expression of iNOS has been reported. This effect of the agent observed here could be presumably attributed to the decreased infiltration of the tissue by leukocytes, the cells which are the main sources of iNOS [17]. In a study by Kane *et al.* [18], iNOS expression was mostly localized in mast cells gathered in angiogenic regions of the flap. These cells were detected to express vascular endothelial growth factor and basic fibroblast growth factor, which contribute to the angiogenesis in the ischemic tissue. Thus, azelaic acid and finasteride presumably induce NO-mediated angiogenesis in the flap tissue and improve survival flap. It is well established that agents with angiogenic activity could improve the outcomes in I/R injury in most tissues [19]. We recommend future investigations for determining the potential role of angiogenesis in improved surgical flap survival mediated by 5α-reductase inhibitors, azelaic acid and finasteride. 

**Fig. 3:**
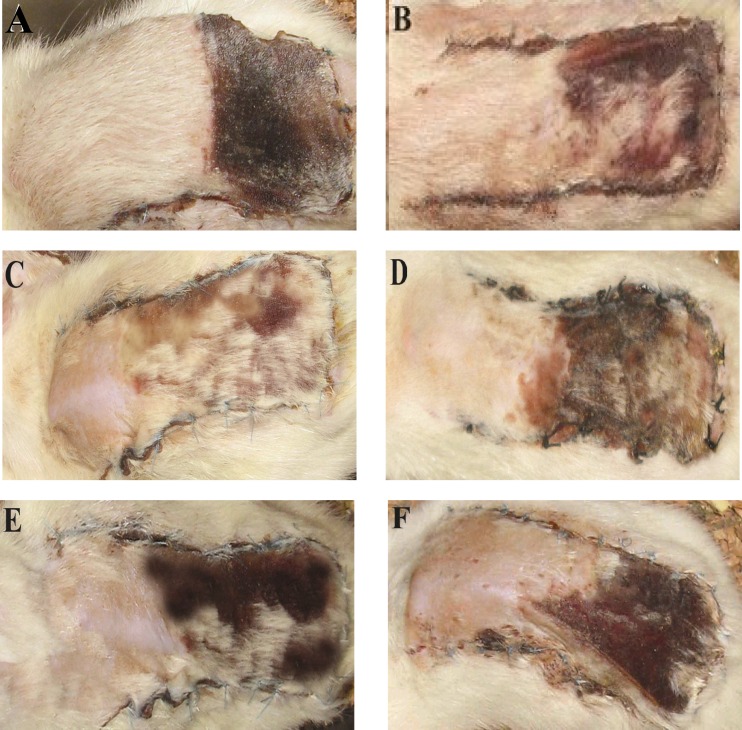
The effect of different types of treatments on necrosis of distal parts of skin flap seven days after surgery. Figures include a sample from each group. (A) The complete necrosis of distal sections in control group, (B) limited necrosis of skin flap in margins of distal parts in finasteride (1 mg/flap, topical application)-treated rats, (C) Decreased necrotic area of flap in topical azelaic acid (100 mg/flap)-treated group, (D) L-NAME (20 mg/kg, i.p.) treatment and extended necrotic area, (E) Pre-treatment with L-NAME in finasteride-treated rats with a large area of distal flap necrosis that has been expanded to the proximal parts, (F) complete necrosis of distal part in L-NAME + azelaic acid-treated group.

**Fig. 4:**
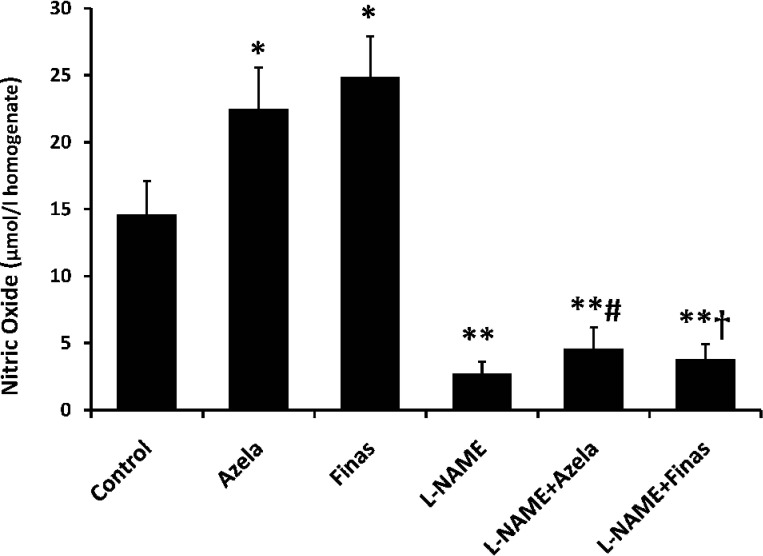
Concentration of NO metabolites in skin tissue homogenate prepared from the flap area. Data are represented as means ± standard error of mean (SEM) of each group. *P < 0.05 and **P < 0.01 vs. control group, #*P* < 0.001 vs. azelaic acid and † *P* < 0.001 vs. finasteride. Azela, azelaic acid; Finas, finasteride.

Figure 3 was taken from the skin flap of one of the rats of each group in which the distal part of the flaps in control and L-NAME-treated group demonstrated extended necrotic area compared to azelaic acid- or finasteride-treated rats. The latter two groups also showed a limited flap necrosis in the margins of distal part. This result further confirms the NO-dependent protective effects of azelaic acid and finasteride treatment in surgical flaps. In control group, total content of NO metabolites was 14.6 ± 2.5 μmol/l tissue homogenate (Fig. 4). Treatment with azelaic acid and finasteride increased nitrite plus nitrate concentrations to 22.5 ± 3.1 μmol/l and 24.9 ± 3 μmol/l homogenate, respectively (*P* < 0.05). L-NAME blocked NO production and decreased NO metabolites to 2.73 ± 0.9 μmol/l (*P *< 0.01). Pre-treatment with L-NAME in azelaic acid and finasteride groups significantly decreased nitrite plus nitrate (4.61 ± 1.6 μmol/l and 3.82 ± 1.1 μmol/l, respectively) in comparison to the control groups (*P* < 0.01), azelaic acid and finasteride-treated groups alone (*P* < 0.001). It appears that azelaic acid and finasteride increase flap survival, at least in part, through induction of NO synthesis via iNOS. However, role of iNOS in protection against I/R injury is a matter of debate. While many studies have shown beneficial effects of iNOS elevation on the I/R injury of the kidney [20, 21], skin [18] and retina [22], some others have reported its detrimental effects on skin [23] and kidney [24].

Beyond the effects of azelaic acid and finasteride on NO activity of the injured tissue, their involvement in alteration of the balance of testosterone 5α-reduction and aromatization may play a role in flap tissue longevity. This result can be deduced from the fact that testosterone 5α-reduction and aromatization are implicated in better response of females to cerebro-vascular and ischemic events [13]. In sum, the results unveiled the beneficial effect of 5α-reductase inhibitors, azelaic acid and finasteride, in surgical flap survival by alterations in NO and iNOS metabolisms. However, further investigations are required to exactly determine the mechanisms of surgical flap survival with these two drugs. 
